# Evaluating Knowledge and Practice Related to Light-Curing and Resin-Based Composites Polymerization

**DOI:** 10.3290/j.ohpd.c_2629

**Published:** 2026-04-13

**Authors:** Afnan O. Al-Zain, Haidar Alhaddad, Rozana A. Al-Bukhary, Dalia E. Meisha

**Affiliations:** a Afnan O. Al-Zain Associate Professor, Restorative Dentistry Department, Faculty of Dentistry, King Abdulaziz University, Jeddah, Saudi Arabia. Conceptualisation, experimental design, data acquisition, data interpretation, drafting the manuscript, critically reviewing the manuscript, approval of the final manuscript, and accountable for all aspects of the work.; b Haidar Alhaddad Researcher, Ministry of Health Clinics, Jeddah, Saudi Arabia. Experimental design, data acquisition, drafting the manuscript, critically reviewing the manuscript, approval of the final manuscript, and accountable for all aspects of the work.; c Rozana A. Al-Bukhary Assistant Professor, Restorative Dentistry Department, Faculty of Dentistry, King Abdulaziz University, Jeddah, Saudi Arabia. Experimental design, data acquisition, data interpretation, drafting the manuscript, critically reviewing the manuscript, approval of the final manuscript, and accountable for all aspects of the work.; d Dalia E. Meisha Associate Professor, Dental Public Health Department, Faculty of Dentistry, King Abdulaziz University, Jeddah, Saudi Arabia. Experimental design, data acquisition, data analysis, data interpretation, drafting the manuscript, critically reviewing the manuscript, approval of the final manuscript, and accountable for all aspects of the work.

**Keywords:** attitudes, dental curing light, dental student, dentists, knowledge, practice, resin composites, survey

## Abstract

**Purpose:**

Delivering high-quality restorative care requires clinicians to possess a solid understanding of light-curing units (LCUs) and resin-based composite (RBC) polymerisation. Despite growing research highlighting knowledge gaps among dental professionals, a disconnect remains between evidence-based recommendations and clinical implementation. This study assessed knowledge gaps and clinical practices related to LCUs and RBC polymerisation among dental students, general dentists, specialists and consultants in Saudi Arabia.

**Materials and Methods:**

A validated, electronic questionnaire collected data on demographics, LCU and RBC selection criteria, knowledge of polymerisation, and related clinical practices.

**Results:**

Among the 291 respondents, 63.9% of participants were unaware of their LCU irradiance, and 52.6% were not aware of the emission spectrum. Related clinical practices, maintenance routines, and infection control were suboptimal, with only 7.9% performed daily irradiance checks and 38.5% lacking a defined maintenance routine. Specialists demonstrated better knowledge than students and general dentists (P < 0.05), yet knowledge gaps persisted across all groups. Notably, 81% of respondents expressed interest in continuing education on LCUs.

**Conclusion:**

Significant deficiencies in LCU selection, photoinitiator compatibility awareness, and maintenance practices were identified across all practitioner categories. Integrating competency-based education and standardised protocols is essential to improve polymerisation quality, ensure long-term restorative success, and safeguard patient care.

The longevity of restorations is influenced by a combination of patient-, dentist-, material-, and light-curing device-related factors, with the dentist’s clinical skills and procedural knowledge playing a critical role.^36,48^ Even high-performance resin-based composites (RBCs) can fail if essential clinical steps, particularly appropriate light-curing unit (LCU) selection and technique, are performed incorrectly, often as a result of inadequate understanding or neglect of photocuring principles.^3,5,33,34,42,67,69^ Although frequently underestimated, these principles are fundamental to achieving durable and successful RBC restorations.^56,59,64,67,74,75^

Effective polymerisation requires various factors, including spectral matching between the LCU and the photoinitiators in the RBC.^44,46,64,67^ Photoinitiators have distinct absorption spectra and are activated at a specific wavelength range.^44,46,64,67^ Different RBCs may incorporate different photoinitiator systems with different spectral requirements.^44,67^ Camphorquinone (CQ), the most widely used photoinitiator, is absorbed broadly in the blue region (~425–495 nm and peaks at ~470 nm).^39,52,60,64,67^ In contrast, alternative initiators such as trimethylbenzoyl-diphenylphosphine oxide (TPO), bis-acylphosphine oxide (BAPO), and 1-phenyl-1,2-propanedione (PPD) are primarily violet-sensitive (~375–425 nm).^39,60,64,66,67^ Accordingly, the contemporary light-emitting-diode (LED) LCUs are either single-emission-peak with blue LED chip/s only (~450–490 nm), effectively activate CQ, or multiple-emission-peak devices incorporate violet LED chip/s (~380–420 nm), in addition to the blue LED chips, enabling activation of alternative photoinitiators, commonly found in aesthetic and bleaching shade RBCs.^8,46,53,56,59,64,67,73^ Therefore, polymerisation efficacy depends on matching the LCU’s emission spectrum with the photoinitiator system of the RBC.^8,9,39,60,64,66, 67^

Failure to achieve adequate polymerisation results in reduced mechanical strength, increased marginal degradation, microleakage, and secondary caries, ultimately compromising long-term clinical outcomes and patient safety.^46,53,56,59,64,67^ This can be a result of inadequate spectral compatibility, poorly controlled curing parameters, including insufficient irradiance, radiant exposure, curing time, incorrect curing technique, and lack of device maintenance, such as decreased irradiance and contaminated or broken light-guide tip, which can result in ineffective activation of the photoinitiator system and yield incomplete polymerisation and inferior properties.^8,53,59,73^ Excessive exposure times exceeding 40 s may elevate pulpal temperature beyond the critical 5.5°C injury threshold, thereby increasing the risk of pulpal damage and compromising pulp vitality, particularly when inappropriate LCU settings are used.^32,47,72,79^ Therefore, careful selection of LCU settings, along with preservation of adequate dentine thickness, minimises this risk.

Worldwide evidence consistently reports significant operator knowledge gaps regarding irradiance, exposure time, spectral compatibility, and maintenance of LCUs, with many clinicians unaware of their device’s output or photoinitiator requirements.^2,15,20,25,30,33,42,55,69^ In the United Kingdom, 84% of orthodontists did not know their LCU’s irradiance, and 67% did not know its wavelength; eye-safety measures were inconsistently used.^51^ A national Norwegian survey found that ~78% were unaware of irradiance, many did not check output, and eye protection was often inadequate.^42^ Additional cross-sectional studies from United States, India, Pakistan, the UK, the United States, Bulgaria, and Brazil report variable maintenance routines, limited conceptual knowledge, inconsistent technique, and suboptimal eye protection, collectively calling for improved education and guidance.^14,23,28,30,31,55,69^

Similar trends have been documented in Saudi Arabia, where inappropriate curing protocols and a lack of maintenance practices are common among practitioners and students.^2,17,18,38,58^ Among general dentists and specialists in Riyadh, many were unsure of LCU type or irradiance and used incorrect terminology; compensatory behaviours (eg, ‘just increase curing time’) were common.^2^ Studies in private clinics and dental schools reported insufficient awareness of routine maintenance, limited familiarity with radiometers, and conceptual gaps in LCU use; national and regional surveys likewise highlighted variability in knowledge, practice, and safety behaviours.^17,18,58^

Despite these well-established principles regarding LCU and RBC polymerisation, most previous studies focused on generic parameters (curing time, distance, angulation, and maintenance) or were typically single-city or targeted specific practitioner level, emphesising limited practice items.^14, 17,18,28,34,42,51,69^ Moreover, no national study in Saudi Arabia has comprehensively evaluated awareness of spectral compatibility, irradiance knowledge, and maintenance practices across all practitioner levels. Identifying these gaps is essential to ultimately improving restoration success, which translates to better patient care.

This study assessed knowledge and clinical practices related to LCU selection, photoinitiator compatibility, and polymerisation protocols among multiple practitioner levels (dental students, general dentists, and specialists/consultants) in Saudi Arabia. The null hypothesis was that no differences would exist among the different practitioners’ strata in knowledge and clinical practice regarding LCU and RBCs.

## MATERIALS AND METHODS

The questionnaire was prepared in the English language and underwent a series of face and content validation steps. The questionnaire instrument was adapted from previous studies,^15,30,42^ and additional original items were included to address the study objectives. It was initially developed by a panel of four dental academics, followed by consultation with three survey methodology experts. Content validation was conducted through consultation with three field experts, whereas face validation involved feedback from five specialist dentists. The questionnaire was then piloted among 10 dentists from the target population, and individual interviews were conducted to gather their feedback. Test-retest reliability was assessed by resending the questionnaire to the same pilot group 2 weeks later.

The final questionnaire consisted of open- and closed-ended items, including multiple-response questions, and was organised into four sections: (1) eligibility and consent; (2) demographics; (3) prior education and sources of knowledge regarding LCUs; and (4) knowledge and clinical practices related to LCU and RBC polymerisation. The clinical practice section addressed LCU selection, use, maintenance, infection control, and eye protection (Supplementary Material 1). The questionnaire took approximately 10 min on average to complete. Data collection took place over a period of 7 months from October to June.

The sample size was calculated based on the most recently reported total number of dentists in Saudi Arabia (27,181).^16^ The calculation assumed a 5% margin of error and 90% confidence level using G-power software. The formula used for sample size calculation was the finite population formula:


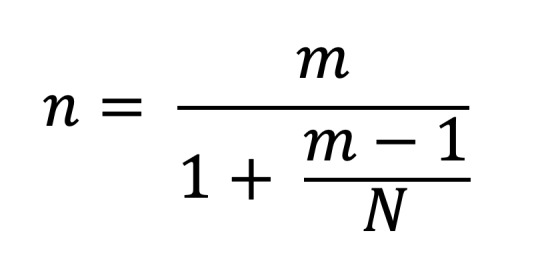


Where:


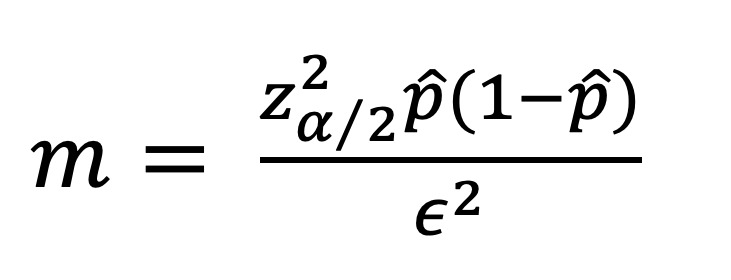


And where:

n = final required sample size (adjusted for finite population)
m = initial sample size estimate for an infinite population
N = total population size (27,181 dentists)
zα/2​ = z-score for 90% confidence level (1.645)
α​ = estimated proportion (0.5)
1−p= complement of the proportion
ϵ = margin of error (0.05)

Based on that, the minimum required sample size was 268 participants.

Statistical analyses were conducted using IBM SPSS Statistics software (version 24; IBM, Armonk, NY, United States). Chi-square tests and Kruskal–Wallis tests with pairwise comparisons using Bonferroni correction were used to assess differences in ranking parameters, knowledge, and clinical practices related to LCUs and RBC polymerisation among the three levels of practitioners (dental students, general dentists, and specialists and consultants) (α = 0.05).

## RESULTS

### Participant Characteristics

A total of 291 participants completed the survey, yielding a response rate of 82.2% (291 out of 354). Table 1 summarises the main characteristics of the respondents. Approximately 57% were female, and the majority were Saudi nationals. Ages ranged from 20 to 62 years, with a mean of 29.1 years.

**Table 1:** Characteristics of the study participants (n = 291)

Characteristic	Overall n = 291	Students n = 100	General dentists n = 94	Specialists/ consultants n = 97
Mean ± SD (Range)				
	% (n)	% (n)	% (n)	% (n)
Age in years	29.1 ± 8.5 (20–62)	22.3 ± 1.6 (20–27)	26.6 ± 5.1 (22–54)	38.6 ± 7.0 (30–62)
Gender				
Male	43.3% (126)	56% (56)	38.3% (36)	35.1% (34)
Female	56.7% (165)	44% (44)	61.7% (58)	64.9% (63)
Nationality				
Saudi	89.7% (261)	99% (99)	93.6% (88)	76.3% (74)
Non-Saudi	10.3% (30)	1% (1)	6.4% (6)	23.7% (23)
Clinical practice				
Full time	46.7% (136)	NA	57.0% (57)	43.0% (43)
Part time	53.3% (155)	NA	40.7% (37)	59.3% (54)
Country of highest obtained educational degree				
KSA	74.5% (214)	100% (100)	93.6% (88)	26.8% (26)
North America	10.7% (31)	–	–	31.9% (31)
Europe and UK	5.9% (17)	–	1.1% (1)	16.5% (16)
Africa and Southeast Asia	6.2% (18)	–	1.1% (1)	17.5% (17)
Far East and New Zealand	1.4% (4)	–	1.1% (1)	3.1% (3)


Of the student respondents (n = 100), participants were distributed across academic years as follows: third year (14%), fourth year (35%), fifth year (33%), and sixth year (18%). The majority of students were enrolled at King Abdulaziz University (KAU) (n = 82), followed by Alfarabi (n = 15), Ibn Sina (n = 2), and Umm Al-Qura University (n = 1). The specialists and consultants who responded (n = 97) had diverse educational backgrounds from various countries. The distribution of these specialists/consultants was as follows: Restorative/Operative Dentistry (n = 46), Prosthodontics (n = 24), Dental Biomaterials (n = 8), Advanced General Dentistry (n = 9), and Orthodontics/Paediatric Dentistry (n = 10). General dentists were more likely to work full-time compared to higher ranks (60.6% vs 41.2%). Although the exact number of years of clinical experience was not collected, the Saudi Commission for Health Specialties (SCFHS) practitioner ranking system provides an indirect measure of clinical experience. Under this system, the designation of specialist or consultant requires completion of postgraduate education and a minimum of 3 years of subsequent clinical practice.

### Knowledge Regarding LCUs and RBC Polymerisation

**Fig 1:**
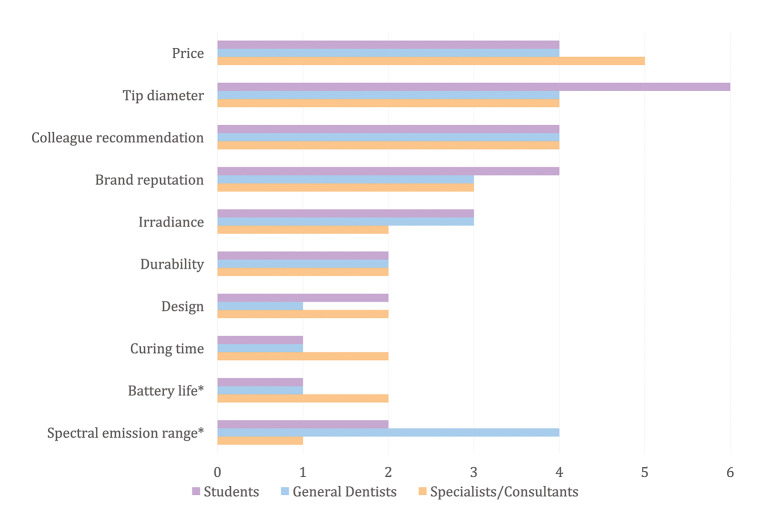
Mean ranking of the parameters used to choose LCU by students, general dentists, and specialists/consultants (1 indicates ranked as first, so a lower value indicates a higher ranking). The top parameters for choosing LCU among the respondents were spectral emission range, curing time and battery life, followed by design. *P value ≤ 0.05

The top three parameters for selecting LCUs among respondents were curing time, battery life, and design, with mean rankings of 4.3, 4.4, and 4.6, respectively. Significant differences were observed among students, general dentists, and specialists and consultants in the ranking of battery life and spectral emission range as selection criteria (Kruskal–Wallis test, P = 0.005; Fig 1). Specialists and consultants ranked spectral emission range as significantly more important than did general dentists and students. In contrast, battery life was considered significantly less important by specialists and consultants compared with the other two groups. Durability and colleague recommendation were rated similarly across all groups, whereas price was ranked as the least important parameter.

**Fig 2:**
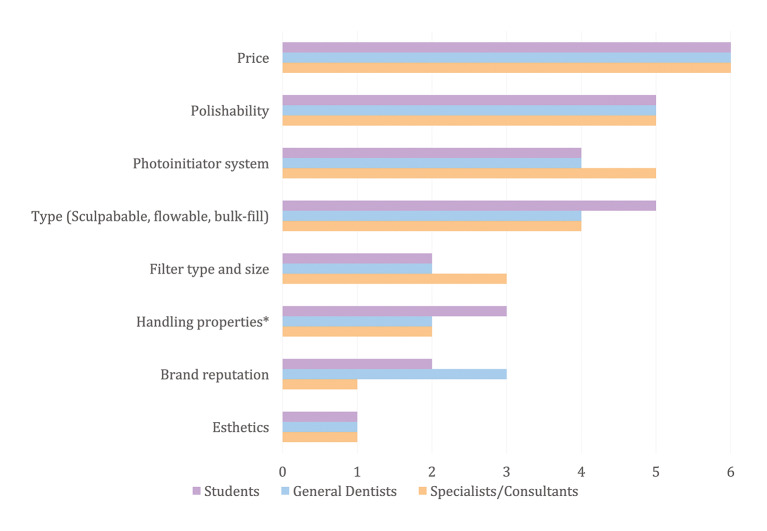
Mean ranking of the parameters used to select an RBC by students, general dentists, and specialists/consultants (1 indicates ranked first, so a lower value indicates a higher ranking). The top parameters for purchaing RBS were ethetic properties followed by brand reputation and ease of handeling. *P value ≤ 0.05

The top parameters for purchasing RBCs were brand reputation, ease of handling, price, and aesthetic properties, with mean rankings of 2.4, 3.4, 3.6, and 3.6, respectively. Among the three groups, significant differences were observed only for handling properties (Kruskal–Wallis test, P = 0.04; Fig 2). Across all groups, aesthetics was ranked as the most important parameter, whereas price was ranked as the least important.

**Fig 3:**
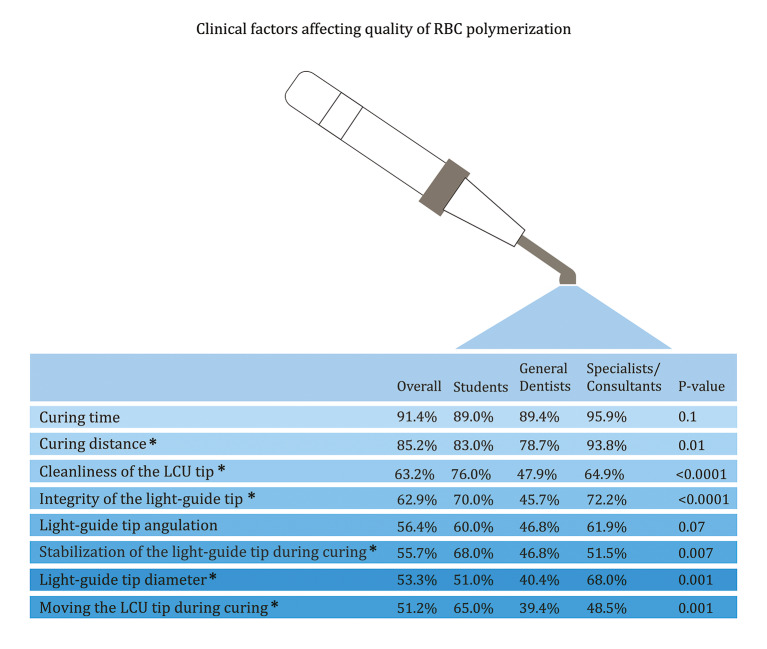
Comparison of the clinical factors affecting the quality of RBC polymerisation among students, general dentists, and specialists/consultants. *P value ≤ 0.05

The survey results revealed significant differences in knowledge regarding LCU use and RBC polymerisation among students, general dentists, and specialists and consultants (Table 2 and Fig 3). A substantial proportion of respondents (52.6%) were unaware of the type of LED used in their LCU. Moreover, 63.9% did not know the irradiance of their device. However, a significantly higher proportion of specialists and consultants (46.4%) reported knowing their LCU irradiance compared with students (30%) and general dentists (31.9%; P = 0.03).

**Table 2:** Comparison of respondents’ knowledge regarding LCU use and RBC polymerisation among students, general dentists, and specialists/consultants

Parameter	Overall n = 291	Students n = 100	General dentists n = 94	Specialists/ consultants n = 97	P value
Type of LED currently using					
Don’t know	52.6% (153)	48% (48)	50% (47)	59.8% (58)	0.2
Irradiance of your LED					
Don’t know	63.9% (186)	70% (70)	68.1% (64)	53.6% (52)	0.03*
Optimum distance for the tip of LCU and RBC					
Correct	75.6 (220)	81.0% (81)	74.5% (70)	71.1% (69)	0.1
Which curing time recommendations should follow					
Correct	75.9% (221)	73.0% (73)	74.5% (70)	80.4% (78)	0.2
How to light cure a large restoration with a small light-guide tip?					
Correct	69.8% (203)	80.0% (80)	60.6% (57)	68.0% (66)	0.001*
LCU effect on pulp					
Correct	51.9% (151)	53.0% (53)	43.6% (41)	58.8% (57)	0.1
LCU effects on eyes					
Correct	93.1% (271)	93% (93)	95.7% (90)	90.7% (88)	0.1
P value of Chi-square tests; (*P-value ≤ 0.05)					

The top clinical variables reported to affect the quality of RBC polymerisation were curing time (91.4%), curing distance (85.2%), cleanliness of the LCU tip (63.2%), and integrity of the light-guide tip (62.9%; Fig 3). A significantly higher percentage of specialists and consultants recognised that curing distance and light-guide tip diameter, along with curing technique steps, influence the quality of polymerisation compared with the other groups (P < 0.01). In contrast, students more frequently recognised the importance of LCU tip cleanliness, stabilisation of the light-guide tip during curing, and moving the LCU tip during curing (P < 0.001). Both specialists/consultants and students more often recognised the impact of light-guide tip integrity on RBC polymerisation compared with general dentists.

**Fig 4:**
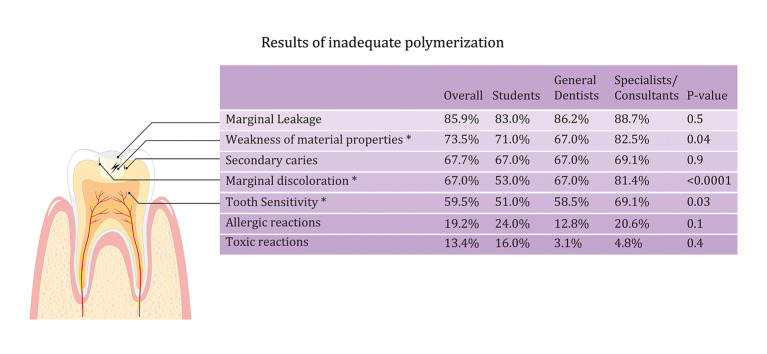
Comparison of the results of inadequate polymerisation among students, general dentists, and specialists/consultants. *P value ≤ 0.05

The most commonly reported consequences of inadequate polymerisation were marginal leakage (85.9%), weakness of material properties (73.5%), development of secondary caries (67.7%), and marginal discolouration (67%; Fig 4). A significantly higher proportion of specialists and consultants identified weakness of material properties, marginal discolouration, and tooth sensitivity as consequences of inadequate polymerisation compared with students and general dentists (P < 0.05).

**Fig 5:**
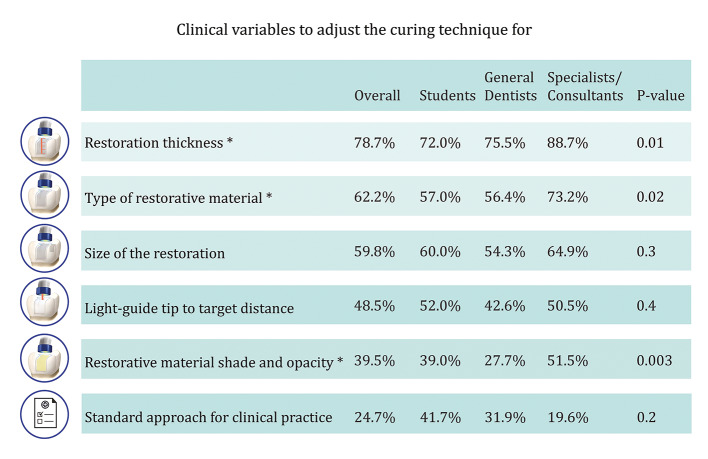
Comparison of the clinical variables considered to adjust for light-curing technique among students, general dentists, and specialists/consultants. *P value ≤ 0.05

The top three reported adjustments to the curing technique based on clinical variables were restoration thickness, type of restorative material used, and size of the restoration. Specialists and consultants reported significantly higher rates of adjusting their curing technique based on restoration thickness, type of restorative material used, and the shade and opacity of the material compared with the other groups (P < 0.01) (Fig 5).

A higher percentage of specialists and consultants (80.4%) correctly recognised that the recommended exposure time should be based on the RBC, not the LCU. In contrast, a significantly higher percentage of students correctly identified the appropriate technique for curing a large restoration with a small light-guide tip – using multiple overlapping irradiation cycles – compared with the other groups (P = 0.001). The effect of LCU use on the pulp was understood by 43.6% to 58.8% of respondents, whereas the effects on the eyes were more widely recognised, with awareness ranging from 90.7% to 95.7%.

### Clinical Practices Regarding LCU and RBC Use

**Fig 6:**
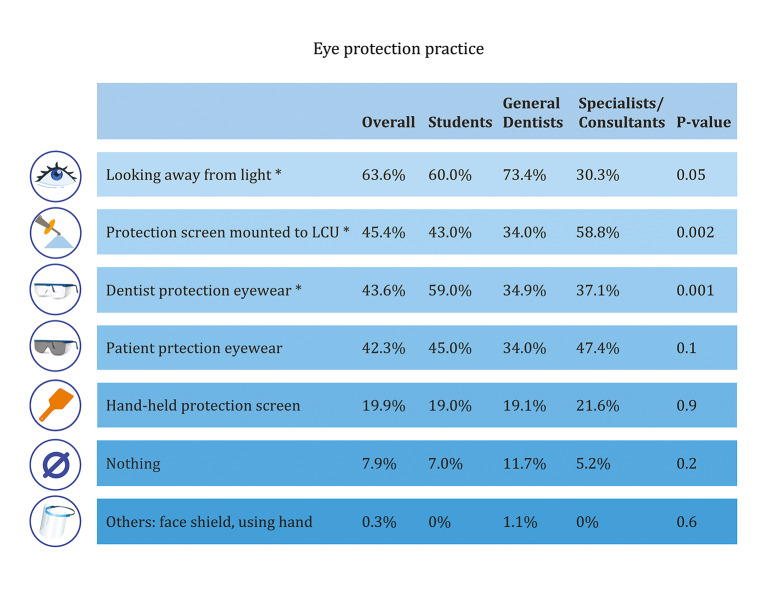
Comparison of the eye-protection practice among students, general dentists, and specialists/consultants. *P value ≤ 0.05

Approximately 54% of dentists reported holding the LCU themselves during the light-curing step, whereas the remainder indicated that dental assistants performed this step. Table 3 outlines practices related to LCU use. A significantly higher percentage of specialists and consultants reported using the LCU more frequently during a typical workday compared with the other groups (P = 0.009). In contrast, significantly more general dentists were unaware of the age of their current LED device (P = 0.02). Regarding eye protection practices, the use of various protective methods ranged from 0.3% to 63.6% (Fig 6). A significantly higher proportion of general dentists (73.6%) and students (60.0%) reported looking away from the light during curing, compared with only 30.3% of specialists and consultants. The use of a protection screen mounted on the LCU was significantly more common among specialists and consultants (58.8%) than among the other groups. Conversely, the use of protective eyewear was highest among students (59%).

**Table 3:** Comparison of the current practice of respondents regarding LCU and RBC use among students, general dentists, and specialists/consultants

Parameter	Overall	Students	General dentists	Specialists/ consultants	P value
	Median	Median	Median	Median	
	% (n)	% (n)	% (n)	% (n)	
Number of times using LCU during a normal working day	4	3	4	5	0.009*
How old is your currently used LCU					
Median number of years (for those who know)	2	2	5	3	0.003*
Don’t know	75.6% (220)	70.0% (70)	80.9% (76)	76.3% (74)	0.02*
Routine for maintenance of LCU					0.006*
No specific routine	38.5% (112)	39.3% (44)	36.6% (41)	24.1% (27)	
Visual control of light-guide tip for scratches, spots, or remains if restorative material	12.0% (35)	6.2% (18)	7.4% (7)	10.3% (10)	
Digital radiometer to monitor irradiance	7.9% (23)	3.9% (3)	6.4% (6)	14.4% (14)	
LCU is regularly checked with dental unit service	6.2% (18)	7.0% (7)	3.2% (3)	8.2% (8)	
Don’t know	35.1% (102)	27.0% (27)	39.4% (44)	39.2% (60)	
Frequency of checking LCU output					0.04*
Daily (recommended)	7.9% (23)	14.0% (14)	5.3% (5)	4.1% (4)	
When the LCU malfunctions	6.9% (20)	9.0% (9)	7.4% (7)	4.1% (4)	
Once per week	6.5% (19)	6.0% (6)	5.3% (5)	8.2% (8)	
Every 1–6 months	6.5% (19)	3.0% (3)	4.3% (4)	12.4% (12)	
Once per year	2.1% (6)	2.0% (2)	1.17% (1)	3.1 (3)	
Don’t check	40.5% (118)	39.0% (39)	48.9% (46)	34.0% (33)	
Don’t know	28.5% (83)	25.0% (25)	26.6% (25)	34.0% (33)	
Checking light-guide tip for debris					0.02*
Before curing	38.8% (113)	45.0% (45)	35.4% (40)	28.9% (28)	
Before and after curing (recommended)	27.5% (80)	34.0% (34)	18.1% (17)	29.9% (29)	
After curing	75.63.8% (11)	4.0% (4)	4.3% (4)	3.1% (3)	
Don’t check	19.9% (58)	11.0% (11)	25.5% (24)	23.7% (23)	
Don’t know	27.510.0% (29)	34.0% (6)	18.1% (9)	29.9% (14)	
Way of cleaning light-guide tip					0.1
Alcohol (recommended)	85.6% (249)	82.0% (82)	84.0% (79)	90.7% (88)	
Water	5.8% (17)	9.0% (9)	7.4% (7)	1.0% (1)	
Fingernail	2.1% (6)	1.0% (3)	0.3% (1)	0.7% (2)	
Sharp instrument	2.1% (6)	4.0% (4)	1.1% (1)	1.0% (1)	
Others: clinic disinfectant, dental assistant not me	4.5% (13)	0.7% (2)	2.1% (6)	1.7% (5)	
Monitoring the battery of a cordless light-emitting-diode (LED) unit?					0.01*
Batteries showing low charge capacity are replaced	25.4% (74)	20.0% (20)	18.1% (17)	38.1% (37)	
Composite samples are periodically test cured	5.8% (17)	7.0% (7)	6.4% (6)	4.1% (4)	
Don’t check	34.0% (99)	31.0% (31)	41.5% (39)	29.9% (29)	
Don’t know	34.0% (99)	42.0% (42)	34.0% (32)	25.8% (25)	
Monitoring the quartz-tungsten-halogen (QTH) unit?					0.06
Bulbs showing low output are replaced	16.5% (48)	12.0% (12)	13.8% (13)	23.7% (23)	
Composite samples are periodically test cured	5.2% (15)	6.0% (6)	6.4% (6)	3.1% (3)	
Don’t check	32.3% (94)	28.0% (28)	41.5% (39)	27.8% (27)	
Don’t know	46.0% (134)	54.0% (54)	38.3% (36)	45.4% (44)	
Infection control practice					
Placing a clear plastic protective sleeve on LCU	67.0% (195)	63.0% (63)	66.0% (62)	72.2% (70)	0.4
Disinfect LCU after every use	61.5% (179)	43.0% (43)	71.3% (67)	71.1% (69)	< 0.0001*
Disinfect LCU but not after every use	6.5% (19)	3.1% (9)	2.1% (6)	4.1% (4)	0.4
Autoclave light-guide tip	5.5% (16)	4.8% (14)	0.3% (1)	0.3% (1)	< 0.0001*
Others	2.4% (7)	1.7% (5)	0.3% (1)	0.3% (1)	0.1
Nothing	6.5% (19)	4.1% (12)	2.1% (6)	0.3% (1)	0.008*
Reading LCU manufacturer instructions					
Yes	48.5% (141)	54.0% (54)	38.3% (36)	52.6% (51)	0.06
P value of Chi-square tests; (*P-value ≤ 0.05)					

Significant differences among the groups were found in routine maintenance of the LCU, frequency of checking LCU output, inspection of the light-guide tip for debris, and monitoring of cordless LED unit batteries. Routine maintenance practices were generally low, ranging from 6.2% to 12%. Furthermore, 38.5% of participants reported not having a specific maintenance routine, and 35.1% did not know what the routine should be. Suboptimal practices were also observed in checking LCU output, as only 7.9% of respondents performed daily output checks as recommended, whereas 40.5% did not check the output at all. Fewer than one-third of participants checked the light-guide tip for debris before and after curing, although most reported cleaning the tip with alcohol (85.6%). Regarding battery monitoring, 34% of respondents did not know how to monitor the battery of their LED LCU, and the same proportion did not know how to check it. For quartz-tungsten-halogen (QTH) units, 46% did not know how to monitor the device, and 32.3% did not perform any monitoring. The most commonly reported infection control practices for LCUs were placing a clear plastic protective sleeve (67%) and disinfecting the unit after each use (61.5%). Specialists and consultants, along with general dentists, were significantly more likely to disinfect the LCU after each use compared with students (P < 0.0001). Significantly more students autoclaved the light-guide tip (4.8%), and 4.1% of students reported not performing any infection control measures, a higher proportion than in the other groups. Fewer than half of all respondents reported reading the instructions of the LCU manufacturer, with general dentists being the least likely to do so.

Approximately 38.8% of respondents believed that they had not received sufficient undergraduate education regarding LCUs. Among those with postgraduate education, 91.7% felt their education on LCUs was also insufficient. The sources used to acquire knowledge about LCUs varied across groups. The top two sources for students were social media (50%) and lectures (45.4%), whereas general dentists most frequently cited webinars (40.5%) and lectures (36.1%). Specialists and consultants primarily relied on postgraduate lectures (73.6%) and conferences (67.9%) for their knowledge. Overall, 81% of respondents expressed interest in attending continuous education courses or lectures on LCUs to enhance patient care.

## DISCUSSION

This survey revealed notable gaps in knowledge and clinical practices related to LCUs and RBC polymerisation among dental students, general dentists, and specialists/consultants in Saudi Arabia. Although specialists demonstrated higher knowledge than students and general dentists, clinically relevant gaps persisted across all practitioner levels, underscoring the need for educational interventions at both undergraduate and continuing professional development levels.

Most participants were senior dental students (fourth–sixth years) after harmonising programme durations across institutions into a six-year framework. Lower participation among third-year students likely reflected their limited prior exposure to light curing, whereas sixth-year students were likely constrained by competing graduation requirements. Although participants were limited to dentists practising in Saudi Arabia, 73% of specialists and consultants graduated from international institutions, reflecting diverse educational backgrounds. As expected, general dentists were more likely to work full-time, whereas specialists and consultants often worked part-time. Our study demographics differed from previous research, as we examined a wider range of strata, including students, general dentists, specialists, and consultants. In contrast, some studies restricted their samples to dental students and interns.^26^ Variations in graduate school programmes are expected, and our findings align to some extent with studies that investigated specialists and consultants who graduated from international institutions, as well as those conducted in different regions of Saudi Arabia or other countries.^2,17^ Other studies focused exclusively on clinicians, with some specifying general dentists or specialists/consultants, but without reporting their graduate school background.^14,15,17,51,70,80^ Furthermore, several studies neither distinguished between general dentists and specialists nor collected information about graduate education.^14,23,42^ These differences in participant demographics and educational backgrounds limit direct comparability across studies and highlight the need to consider contextual and institutional variations when interpreting findings.

The null hypothesis was rejected, confirming significant differences in LCU and RBC selection parameters among practitioner groups. These findings highlight how differences in background and clinical focus may shape LCU decision-making. Although general dentists and students considered curing time and battery life, and general dentists additionally emphasised design as critical parameters when selecting an LCU, spectral emission was not prioritised among the most important factors. This may suggest limited awareness of the significance of spectral compatibility between the LCU and the photoinitiator, which could impact the effectiveness of polymerisation and overall clinical outcomes.^45^ The higher prioritisation of spectral emission range by specialists and consultants compared with the other groups indicates their awareness of its role in achieving optimal RBC polymerisation. These results were similar to a previous study where students were less knowledgeable than specialists and consultants in this area, and only 11% of total respondents identified spectral emission range as a top selection factor, highlighting a persistent knowledge gap.^7^ The broad-spectrum LCUs, such as QTH or multi-emission-peak LED units, are particularly needed for curing RBCs with unknown or mixed photoinitiators, including camphorquinone and alternative photoinitiators.^2,8,9,13,29,53,59,61,65,73^ This underscores the importance of aligning the LCU emission spectrum with RBC photoinitiators to ensure clinical success.

The consistently low prioritisation of the photoinitiator system when selecting RBCs across all groups highlights a fundamental knowledge gap regarding LCUs and RBCs compatibility. This is concerning, as compatibility between the LCU emission spectrum and RBC photoinitiators is essential for effective polymerisation and restoration longevity, as outlined by the Grothaus–Draper law (First Law of Photocuring).^40^ This disconnect between theoretical knowledge and clinical application underscores the need for clearer manufacturers’ labelling and enhanced professional education.^38^

Our results showed that battery life and curing time were a higher priority among students and general dentists, likely reflecting practical concerns such as workflow efficiency.^74,75^ However, this preference may be influenced by convenience rather than evidence-based decision. Inappropriate emphasis on shortening the curing cycle without consideration of the spectral output may compromise polymerisation quality.^10,12,46,59,64,67^

Although brand reputation was acknowledged for perceived reliability across groups, it was not ranked among the top selection criteria. Our results were similar to previous work.^7^ However, relying solely on brand reputation may lead to overlooking objective parameters may limit evidence-based decision-making.^11,60,64,74,75^

The low prioritisation of price suggests a willingness to invest in LCUs that optimise clinical outcomes, reflecting a belief that quality and efficiency should not be sacrificed for short-term savings. Selecting budget LCUs, which are low-cost devices purchased online, raises concerns about quality, as they frequently exhibit inconsistent performance, poor durability, and incomplete user documentation.^5,11,19,74,75^

When selecting an RBC, handling properties were significantly more important to specialists and consultants than to other groups, although they were generally considered less critical than aesthetics. The prioritisation of aesthetics, brand reputation, and handling properties as the top three factors, along with the consistently low ranking of price, reflects a strong commitment to high-quality, visually appealing restorations despite higher costs. The low emphasis on price further highlights a willingness to invest in premium materials to ensure long-term durability and superior results. This preference, shared across all groups, underscores a collective focus on optimal clinical outcomes and patient satisfaction. Nevertheless, none of the groups prioritised the photoinitiator system as one of the top three parameters to consider, underscoring the knowledge gap regarding sufficient polymerisation quality and better longevity.

A substantial proportion of respondents were unaware of their LCU irradiance, including 70% of students and 53.6% of specialists and consultants, a critical determinant of polymerisation effectiveness and degree of conversion. Inadequate polymerisation has been associated with marginal leakage, inferior physical properties, and secondary caries, and potentially increased risk of restoration failure, consistent with findings from previous studies.^10,12,13,46,59,64,67,74,75^ Other studies have supported this finding, where high percentages of participants lacked knowledge of the irradiance of the LCU they are using.^2,7,15,42,51^

Our survey showed that respondents recognised common consequences of inadequate polymerisation, including marginal leakage, material weakness, and secondary caries. However, a significant awareness gap was noted between students and specialists regarding issues such as marginal leakage, marginal discolouration, and tooth sensitivity. Notably, only 19.2% and 13.4% of respondents identified allergic and toxic reactions, respectively, indicating a critical knowledge deficit. Studies have shown that insufficient polymerisation leads to the release of unreacted monomers and free radicals into the oral environment, compromising restoration integrity and longevity.^1,43,45,46,50,57^ It also promotes increased Streptococcus mutans biofilm formation and surface roughness, elevating the risk of recurrent caries around restorations.^50^ Other studies showed similar findings.^15,17, 34,55,58,70^

Participants correctly identified several clinical factors that influence RBC polymerisation effectiveness, based on the literature.^46,64,67^ Respondents recognised key factors influencing polymerisation such as curing time, distance, and LCU tip cleanliness. Specialists, compared to the other two groups, were particularly more likely to adjust curing protocol based on restoration thickness, material type, and shade, reflecting deeper experience and understanding of complex cases. Our results were similar to those of other studies.^28,31,34,46,49,55,64,67,69^

Significant gaps were found in LCU use and maintenance. Notably, 44–49% of participants were unaware of the impact of light-guide tip stabilisation, angulation, and diameter on polymerisation effectiveness, as well as the effect of LCU output on the pulp. Students, however, showed better understanding of using multiple irradiation cycles for large restorations with small light-guide tips, reflecting a focus on tailored curing methods consistent with prior research.^46,56,59,64,67,74,75^ Other studies supported our findings.^2,18,23,26,28,55,70^ These results highlight areas that could be effectively addressed using educational tools such as the Managing Accurate Resin Curing–Patient Simulator (MARC-PS; BlueLight Analytics, Halifax, Canada). The MARC-PS system provides real-time, objective feedback on key factors influencing polymerisation, such as tip angulation, distance, and stability, and has been shown in previous studies to significantly improve light-curing skills, enhance technique accuracy, and increase operator confidence.^4,6,20,22,24,27,35,37,41,49,63,68,71,76–78^ Although the present survey did not involve direct MARC-PS training, the gaps identified, particularly in tip positioning and stabilisation, are precisely the competencies this tool is designed to strengthen. Integrating MARC-PS training into undergraduate and continuing education programmes could therefore be a targeted approach to address the deficiencies revealed in this study.^6,20^

The study revealed significant inconsistencies in LCU maintenance practices, particularly among students and general dentists, despite evidence emphasising the importance of regular irradiance checks and tip cleanliness.^21,64,67,74,75^ Many participants did not know the age of their LED LCU. This is concerning, given the decline in performance over time without proper maintenance.^11,15,21,56,74,75^ The literature recommends using a dental radiometer to check irradiance output daily before clinical sessions.^74,75^ However, only 7.9% of respondents followed this practice. Moreover, daily irradiance check or checking the LCU irradiance before every clinical session is critical to ensure the needed irradiance is received by the restoration for effective polymerisation.^74,75^ Only 6.2–12% reported having a defined maintenance routine. This can compromise the longevity of restoration and pulp health due to inadequate polymerisation.^11,56,64,67,74,75^

Infection control practices, such as using protective sleeves and disinfecting LCUs, were more commonly followed, although inconsistencies remain. Addressing these gaps requires integrating maintenance protocols and infection control guidelines into dental education and clinical practice to ensure LCU reliability, effectiveness, and longevity. Other studies supported our findings.^15,21,56,64,67^

Eye protection practices varied widely (19.9% to 63.6%), with students more likely to wear protective eyewear than general dentists and specialists. Many respondents relied on physical barriers or looking away instead of appropriate filtered eyewear. This reflects limited awareness of blue light hazards,^54,62,64^ as it risks long-term retinal damage.^54,62,64^ Other studies supported our findings.^2,15,18,23,26,28,55,70^ These results underscore the need for training on occupational safety measures. Equally important is establishing a well-defined maintenance protocol to ensure consistent and effective curing outcomes. Therefore, it is strongly recommended that dental institutions and clinical practices develop and disseminate such protocols, while reinforcing adherence through training and supervision, to safeguard both clinicians and patients.

Approximately 54% of dentists reported holding the LCU themselves during curing, whereas the remainder delegated the task to a dental assistant. Delegation without proper training risks suboptimal polymerisation owing to incorrect angulation or distance, an issue supported by a previous study.^38^

The study revealed widespread dissatisfaction with existing educational exposure regarding LCUs and demonstrated a strong interest in further training (81%) highlights the demand for improved education in this topic.

Numerous studies, including our own, have demonstrated a persistent deficiency in knowledge and clinical practice regarding LCUs among dental students, interns, and practitioners. This highlights a critical gap that requires urgent attention to ensure that theoretical knowledge is effectively translated into clinical competence. Previous research has shown that dentists, dental interns, and dental students prefer learning about light-curing primarily through hands-on workshops, followed by face-to-face lectures rather than online modalities.^7^ Concise, evidence-based multimedia videos delivered via reputable university or professional association platforms were also valued as supplemental resources.^7^ These findings underscore the need for structured, competency-based continuing education programs to bridge knowledge gaps and enhance clinical effectiveness and patient safety. Consistent with existing literature, the results highlight a critical need for continuing education courses to address these gaps and equip dentists with the skills needed to enhance clinical effectiveness and patient safety.^2,11,25,42,59,69,76^

Strengths of this study include its large national sample and inclusion of practitioners across all training levels. However, the study is limited by self-reported data, which may introduce social desirability bias. However, the reported knowledge and practice deficiencies suggest that this bias is minimal. Another limitation is that participants were limited to dentists practising in Saudi Arabia, although the educational diversity of respondents reduces concerns about generalisability. The use of convenience snowball sampling is also a limitation.

These findings reveal significant deficits in knowledge and implementation of optimal LCU procedures across all professional groups, affecting both restorative outcomes and practitioner safety. Deficiencies were evident in understanding curing parameters, recognising the importance of LCU–RBC photoinitiator compatibility, performing routine irradiance checks, and maintaining equipment. Suboptimal infection control and inconsistent use of protective measures were also noted. Addressing these gaps requires coordinated efforts from dental schools, continuing education providers, regulatory bodies, and manufacturers to ensure that clinicians receive structured, evidence-based training through various delivery methods and platforms, and have access to reliable equipment and guidance.

To translate these findings into practice, we propose the following specific recommendations aimed at improving clinical proficiency, standardising best practices, and ultimately enhancing patient care.

### Recommendations

Based on the findings of this study and supported by evidence from previous research, the following actions are recommended to address knowledge and practice gaps related to LCUs and RBC polymerisation:

1. Integrate targeted LCU and RBC education into undergraduate curricula: Include dedicated modules or lecture details on LCU selection parameters, RBC photoinitiator compatibility, curing techniques, and device maintenance. Support this education with patient simulators and hands-on workshops that provide real-time feedback on curing techniques.
2. Enhance continuing professional development with multimedia support: Offering short, focused in-person lectures and practical workshops tailored for practising clinicians, scheduled to fit busy clinical workloads and focus on high-impact skills. Supplement these with concise, peer-reviewed instructional videos hosted on reputable platforms such as university or professional association YouTube channels to reinforce concepts and reduce reliance on unverified sources.
3. Mandate irradiance monitoring and maintenance protocols: Provide access to calibrated dental radiometers in all clinical settings, require daily output checks before clinical sessions, and maintain periodic maintenance logs for all LCUs.
4. Promote manufacturer transparency and informed purchasing: Require clear labelling of LCU spectral emission ranges and RBC photoinitiator types on packaging, and educate clinicians on interpreting this information to ensure spectral compatibility.
5. Standardise infection control and eye safety: Enforce protocols for protective sleeves, disinfection after each use, and use of certified blue light–blocking eyewear during curing.
6. Evaluate the impact of training on clinical outcomes: Conduct longitudinal studies to assess whether improved knowledge and practices translate into enhanced restoration longevity, polymerisation quality, and patient satisfaction.

## CONCLUSION

Substantial deficiencies were identified in the knowledge and implementation of evidence-based clinical practices related to LCUs and RBCs. While specialists demonstrated greater awareness than other groups, deficiencies were evident across all professional levels. Addressing these gaps requires integration of competency-based education and standardised clinical protocols for polymerisation practices, and LCU handling and maintenance. Implementing these measures will enhance restorative outcomes and safeguard patient care.

### Acknowledgements

This project was funded by the Deanship of Scientific Research (DSR) at King Abdulaziz University, Jeddah, under Grant No. (GPIP: 1201-165-2024). The authors, therefore, acknowledge with thanks DSR for technical and financial support. The authors would like to thank all participants who completed the questionnaire. Special thanks go to Deema Meisha for assistance with the infographics. During manuscript preparation, the authors used ChatGPT and Gemini to improve writing and readability.

#### Declaration of interest

The authors declare no conflicts of interest.
